# PP4 deficiency leads to DNA replication stress that impairs immunoglobulin class switch efficiency

**DOI:** 10.1038/s41418-018-0199-z

**Published:** 2018-09-20

**Authors:** Ming-Yu Chen, Wei-Chan Hsu, Shu-Ching Hsu, Yu-Shao Yang, Tsung-Hsien Chuang, Wen-Jye Lin, Tse-Hua Tan, Yu-Wen Su

**Affiliations:** 1 Immunology Research Center, National Health Research Institutes (NHRI), Zhunan, Miaoli County Taiwan; 2 National Institute of Infectious Diseases and Vaccinology, NHRI, Zhunan, Miaoli County Taiwan; 3 Graduate Institute of Medicine, College of Medicine, Kaohsiung Medical University, Kaohsiung, Taiwan; 4 Department of Medical Research and Development, Chang Bing Show Chwan Memorial Hospital, Chang Hua, Taiwan; 5 Department of Pathology & Immunology, Baylor College of Medicine, Houston, TX USA

**Keywords:** B cells, Immune cell death

## Abstract

The serine/threonine phosphatase PP4 has been implicated in DNA damage repair and cell cycle regulation through its dephosphorylation of specific substrates. We previously showed that PP4 is required for mouse B cell development, germinal center (GC) formation and immunoglobulin (Ig) class switch recombination (CSR). Here, we investigate the mechanisms underlying this requirement and demonstrate that murine PP4-deficient B lymphocytes have a defect in cell proliferation. Strikingly, the DNA damage response pathway that involves ATM/p53 and is linked to cell cycle arrest and impaired cell survival is strongly induced in these mutant B cells. In response to LPS + IL-4, stimuli that trigger IgG1 production, these PP4-deficient B cells show inefficient phosphorylation of ATR, leading to reduced retention of γH2AX-NBS1 complexes at sites of DNA damage, and compromised switching to IgG1. However, beyond the cell proliferation phase, conditional deletion of PP4 under the control of AID/cre completely restores normal IgG1 production in mutant B cell cultures. In vivo, co-deletion of PP4 and p53 by AID/cre partially rescues switching to IgG1 in B cells of mice immunized with TNP-KLH. Our findings establish that PP4 is indispensable for preventing DNA replication stress that could interfere with CSR, thereby promoting antibody switching during the humoral immune response.

## Introduction

In response to antigen challenge, naïve B cells become activated, proliferate and form a germinal center (GC), which is essential for the induction of an adaptive humoral immune response. During GC formation, B cells with surface expression of immunoglobulin (Ig) of subclass IgM undergo class switch recombination (CSR), in which the gene segment encoding the Ig constant (C) μ region is excised from the DNA and replaced with one of the available 3’ Ig constant regions Cγ, Cε, or Cα. This process is catalyzed by activation-induced cytidine deaminase (AID) via conversion of cytosines to uracils in the S regions located upstream of each of the C regions [1]. The uracil bases are subsequently processed by uracil DNA glycosylase (UNG) and apurinic/apyrimidinic endonuclease (APE), resulting in DNA double-strand breaks (DSBs) at the donor and the acceptor S regions [2]. The presence of DSBs in DNA activates ATM kinase to phosphorylate p53 and other substrates, resulting in cell cycle regulation and/or apoptosis [3]. During DNA DSB formation, H2AX is immediately activated and phosphorylated at S139 by ATM or ATR to form phosphorylated H2AX (also called γH2AX). γH2AX subsequently recruits DNA damage molecules such as the MRN complex, composed of Mre11, Rad50, and NBS1, which recruits and activates ATR for DNA repair [4–8]. During DNA damage and DNA replication, replication protein A (RPA) complex consisting of RPA1, RPA2, and RPA3 is recruited to single-stranded lesions in DNA, that stabilizes stalled replication fork and facilitates the activation of ATR signaling [9, 10]. To complete CSR, the broken S regions in the Ig loci are re-joined by the homologous recombination (HR) pathway or the classical nonhomologous end-joining (NHEJ) pathway [11]. During such DNA repair, γH2AX functions synergistically with NBS1 [12, 13] to maintain genomic stability [14–16]. In addition, both γH2AX and NBS1 are required to complete Ig class switching [17–19]. Once CSR is successful, the B cells produce IgG, IgE, or IgA antibodies whose effector functions confer comprehensive immune protection [20].

Protein phosphatase 4 (PP4) is a well-conserved holoenzyme composed of a catalytic subunit termed PP4c and multiple regulatory subunits. Through its dephosphorylation of various substrates, PP4 has been implicated in microtubule organization [21, 22], histone modification [23], and signal transduction [24–28]. Furthermore, PP4 plays essential roles in DNA damage responses and DNA repair by dephosphorylating DBC1 [29] and KAP1 [30–32], and regulates cell cycle progression by dephosphorylating γH2AX and RPA2 [33–36]. Lastly, several lines of evidence support a crucial function for PP4 in cell proliferation [37–40].

We have previously shown using B cell-specific PP4-deficient mice that B cell development and GC formation are impaired in the absence of this phosphatase and that CSR efficiency is reduced [41, 42]. However, due to the multiple physiological roles of PP4 within mammalian cells, the precise molecular mechanism regulated by PP4 during CSR was unclear. In this study, we utilized CD23/cre or AID/cre to delete the PP4 gene conditionally in mature mouse B cells or activated GC B cells, respectively, to study the effect of PP4 deficiency specifically on CSR. We found that, in response to treatment with lipopolysaccharide plus interleukin-4 (LPS + IL-4), B cells in which PP4 was deleted under the control of CD23/cre underwent cell cycle arrest in S phase. These mutant B cells suffered severe DNA damage as revealed by their vigorous activation of the ATM-p53 pathway. In the early phase in response to LPS + IL-4, ATR was not efficiently phosphorylated in these B cells and fewer γH2AX-NBS1 complexes were retained at sites of DNA damage. In contrast, deletion of PP4 by AID/cre completely rescued B cell proliferation and restored normal levels of switching to IgG1 in vitro. Co-deletion of PP4 and p53 by AID/cre led to a partial rescue of IgG1^+^ B cells in vivo in mice immunized with TNR-KLH. Our findings reveal that PP4 is crucial for CSR due to its function in preventing DNA replication stress.

## Materials and Methods

### Mice

PP4^F/F^ mice [24], CD23/cre mice [43], AID/cre mice [43], and p53^F/F^ mice (Jackson lab Stock 008462) [44] generated as previously described were maintained in the Laboratory Animal Center of the National Health Research Institute (NHRI). The CD23/cre mice and AID/cre mice used in all experiments were heterozygous. Double mutant mice were generated in-house using standard breeding procedures. Age-matched mice (8–12 weeks old) were used in all experiments. All mice were euthanized by carbon dioxide inhalation. The mouse numbers for each experiment are specified in the Figure Legends. All animal studies were reviewed and approved by NHRI’s Institutional Animal Care and Use Committee (Permit Number: 099111-A, 103075-A, and 105033-A), and all efforts were made to minimize mouse suffering.

### B cell purification and cell culture

Single cell suspensions were prepared as described previously [42]. In brief, non-B cells were depleted using a cocktail of biotin-conjugated Abs recognizing NK1.1, Thy1.2, Gr1, CD11b, CD11c, or Ter119 (BD Biosciences), followed by application of IMag^TM^ Streptavidin Particles Plus beads (BD Biosciences). B cells of purity between 96–98% were cultured in RPMI 1640 medium supplemented with 2 mM L-glutamine (Gibco), 100 U/ml penicillin plus 100 μg/ml streptomycin (Gibco), 10 mM Hepes (Gibco), 1 mM sodium pyruvate (Gibco), 0.055 mM 2-mercaptoethanol (Gibco), and 10% FBS (Hyclone) (maintenance medium).

### Proliferation assays

For CFSE decay or VPD experiments, B cells were seeded at a density of 2 × 10^7^ in 1 ml PBS and incubated with 5 µM carboxyfluorescein succinimidyl ester (CFSE, Sigma) or 1 µM Violet Proliferation Dye 450 VPD450 (BD Horizon™) at room temperature (RT) for 10 min with gentle mixing. To stop labeling, 10 ml 10% maintenance medium was added to cells followed by centrifugation at 1500 rpm for 10 min. Cell pellets were suspended in PBS or maintenance medium and used for experiments. For BrdU incorporation, B cells were seeded at a density of 1 × 10^6^/ml (without stimulation), cultured in maintenance medium containing 10 µM BrdU (Sigma) for 72 h, and analyzed using the BrdU Flow Kit (BD Pharmingen^TM^ 552598) following the manufacturer’s instructions.

### Induction of CSR

B cells were seeded at a density of 1 × 10^6^/ml and cultured for 1 to 6 days in maintenance medium containing either 10 μg/ml LPS (InVivo Gene) to induce switching to IgG3, or in maintenance medium containing 10 μg/ml LPS plus 20 ng/ml IL-4 (PeproTech) to induce switching to IgG1. B cells with surface expression of IgG1 or IgG3 were identified by flow cytometric analysis of the IgM^-^IgD^-^ gated population as described below.

### Flow cytometric analyses (FACS)

Single cell suspensions of 1 × 10^6^ cells were washed twice with FACS buffer (2% BSA/PBS, 0.01% NaN_3_) and maintained in the dark at 4 °C throughout experiments. Flow cytometric data were acquired using a CantoII flow cytometer and FACSDiva software (both from BD Biosciences). FlowJo software (Tree Star, Inc.) was used for data analyses. For marker expression determinations, cells were incubated for 15 min on ice with anti-mouse antibodies recognizing IgG1 (RMG1–1)-FITC/BV510, CD138 (281–2)-PE (all from eBioscience), B220 (RA3–6B2)-APC/APC-Cy7, IgD (11–26 c.2a)-FITC/PE/Pacific blue/BV510, IgM (II/41)-FITC/APC/PE-Cy7, IgG3 (R40–82)-biotin, or streptavidin-APC (all from BD Biosciences).

For viability determinations, immunostained cells were washed twice in FACS buffer prior to incubation with 7AAD (Sigma). Viable cells were gated from the 7AAD-negative population prior to analysis. For intracellular staining, cells were fixed and permeabilized using the Fixation/Permeabilization solution kit (BD, Cat. NO. 554714) following the manufacturer’s instructions, and stained with anti-p-ATM S1981-PE (10H11.E12, Millipore).

### Immune response in vivo

To examine immune responses and class switching in vivo, mice were i.p. immunized with 5 µg/g body weight of the T-dependent antigen TNP-Keyhole Limpet Hemocyanin (TNP-KLH, BIOSEARCH) suspended in alum (Sigma). Spleen samples were harvested on day 0 and day 14 post-immunization, and IgG1 switching was analyzed by flow cytometry as described above.

### Retroviral transduction

Retroviral vector pMSCV-PIG (Puro-IRES-GFP) empty vector [45] was a gift from David Bartel (Addgene plasmid # 21654). To express genes of interest by retroviral system, genes encoding PP4 WT, PP4 R236L, p53 WT, p53 S15A, or p21 were cloned into the multiple cloning sites of pMSCV-PIG. Retroviral vectors were transfected into Phoenix cells using calcium phosphate, and supernatants containing virus particles were harvested after 48 h. Prior to transduction, purified B cells were seeded at 1 × 10^6^/ml in 24-well plates in maintenance medium containing 10 μg/ml LPS plus 20 ng/ml IL-4 and incubated for 24 h. To infect stimulated B cells, the culture medium was replaced by 750  µl viral supernatant containing 8 μg/ml protamine sulfate (Sigma), and cells were centrifuged at 2500 rpm for 90 min. Transduced cells were cultured for another 48 h in maintenance medium before evaluation of GFP and IgG1 expression by FACS analysis.

### RT-PCR

To examine levels of germline and class-switched Ig transcripts, PCR oligomer primers and reaction conditions were employed as previously reported [46]. PCR reactions were performed using mRNA extracted from B cells that had been left unstimulated, or stimulated with LPS alone, or with LPS + IL-4 for 1 to 4 days, as specified in the text. The mRNA levels revealed by agarose gel electrophoresis were quantified by Image J and normalized to the level of *hprt* expression. The expression level of each transcript in CD23/cre;PP4^+/+^ control mice was defined as 1.

### Cytosolic/nuclear extraction and immunoblotting

Protocol to separate cytosolic and nuclear for immunoblotting was as previously reported [47]. In brief, B cells (1 × 10^7^) were lysed in 20 µl buffer A (10 mM Hepes, 10 mM KCl, 1.5 mM MgCl_2_, 0.34 M sucrose, 10% glycerol, 1 mM DTT, 0.1% Triton X-100, with protease inhibitor freshly added) and left on ice for 5 min. The lysate was centrifuged by 1300 × *g*, 4 °C for 4 min to receive nuclei pellet. The supernatant was further clarified by 20,000 × *g*, 4 °C for 15 min to remove cell debris and insoluble aggregates and receive the cytosolic fraction. Nuclei were washed once in buffer A, and then lysed in 30 µl buffer B (3 mM EDTA, 0.2 mM EGTA, 1 mM DTT, and freshly added protease inhibitors). After centrifugation by 1700 × *g*, 4 °C for 5 min, the yielded supernatant as nuclear fraction was collected. In-house anti-PP4 antibody was generated as described previously [42]. Antibodies recognizing p-p53 S15 (#9284), p-ATR S428/S431 (#2853), p-Chk1 S345 (#2348), p-NBS1 S343 (#3001), NBS1 (#14956), γH2AX (#9718), or H2AX (#7631) were from Cell Signaling. Antibodies recognizing ATM (2C1), p-ATM S1981/1987 (10H11.E12), GAPDH (GTX100118), or p84 (5E10) were from GeneTex; and anti-p53 (SC-6243) was from Santa Cruz. ECL Plus^TM^ Western Blotting Detection Reagents (Amersham) were utilized for immunoblot development.

### Confocal microscopy images

For immunofluorescence staining, B cells were fixed and permeabilized as described above. Permeabilized cells were stained on ice for 1 h with anti-p53-Alexa 488 (Cell Signaling #2015), anti-RPA1-Alexa 647 (abcam #199240), anti-γH2AX-Alexa 647 (Cell Signaling #9720), or with unconjugated antibodies recognizing NBS1 (Cell Signaling #14956) or p-p53 S15 (Cell Signaling #9284). A secondary incubation with goat anti-rabbit-Alexa 488 (Invitrogen #A11034) on ice for 1 h was then performed. After washing, cells were mounted onto slides using ProLong^TM^ Gold Antifade Mountant with DAPI (Invitrogen, P36935). Immunofluorescence was detected by confocal microscopy (Leica TCS SP5 II). Parameters such as fluorescence intensity, focus counts, and co-localization were counted by MetaMorph^®^ software.

### Statistical analysis

Data were analyzed using a one-tailed distribution, type 3 Student’s *t*-test. Differences between treatment groups with *P*-values ≤ 0.05 were considered statistically significant. Symbols of p-values were defined as: *p ≤ 0.05; **p ≤ 0.005 and ***p ≤ 0.0005.

## Results

### Deletion of PP4 by CD23/cre impairs B cell division and viability

**Fig. 1:**
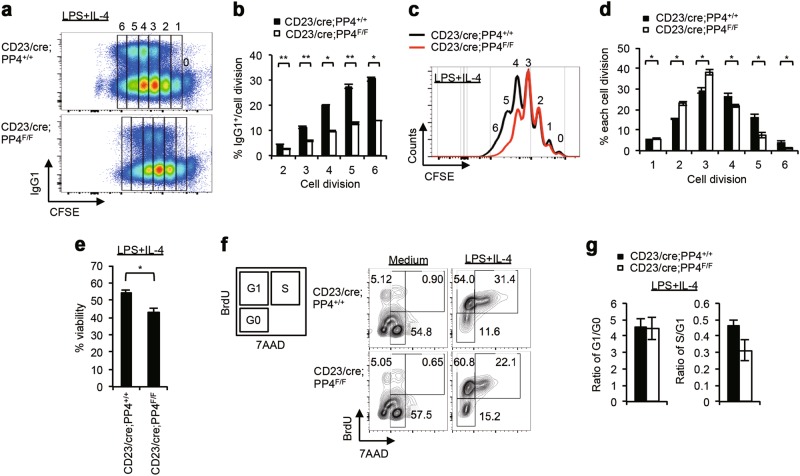
Reduced Ig class switch efficiency is associated with impaired cell division in PP4-deficient B cells. **a** FACS profiles of CFSE decay versus IgG1 expression by B220^+^IgM^-^IgD^-^ gated B cells that were isolated from mice of the indicated genotypes (*n* = 3/group) and stimulated with LPS + IL-4 for 72 h in vitro. Numbers 0 to 6 indicate cell divisions. Data are representative of three independent experiments. **b** Quantitation of frequencies of IgG1^+^-switched B cells after the 2nd to 6th cell divisions among the B220^+^IgM^-^IgD^-^ gated B cells in the experiment described in **a**. Data are the mean ± SD of triplicates. *p ≤ 0.05; **p ≤ 0.005. **c** Overlay of CFSE decay curve and proliferation curve as determined by FACS for the proliferating B cells in **a**. Data are representative of two independent trials. **d** Quantitation of the percentage of proliferating cells in the 1st to 6th cell divisions of the B cells in **a**. Data are the mean ± SD (*n* = 3/group). **e** Quantitation of the percentage of viable B cells stimulated with LPS + IL-4 for 72 h in **a**. Data are the mean ± SD (*n* = 3/group). **f** Left: Schematic illustration n of cell cycle phases in a FACS profile. Right: FACS profiles showing BrdU incorporation by purified B cells of the indicated genotypes that were stimulated with LPS + IL-4 for 72 h in vitro. Numbers in quadrants are frequencies of BrdU/7AAD-positive cells in each phase, as indicated. Data are representative of three independent trials. **g** Quantitation of the ratio of cells in G1 versus G0 phase (left) or in S versus G1 phase (right) in the experiment described in **f**. Data are the mean ± SD (*n* = 3/groups)

We previously showed that B cells from CD23/cre;PP4^F/F^ mice exhibited impaired B cell receptor (BCR) signaling and reduced CSR efficiency [42]. To examine PP4’s involvement in CSR at the molecular level, we isolated splenic B cells from control CD23/cre;PP4^+/+^ (wild type; WT) and mutant CD23/cre;PP4^F/F^ mice, stimulated these cells in vitro with LPS + IL-4 for 72 h to induce IgG1-switching, and concomitantly subjected these cells to CFSE labeling to monitor cell division. As expected, the frequency of IgG1^+^ B cells in the control culture increased gradually to 30% over the 2nd to 6th cell divisions (Figs. 1a and 1b). In the mutant culture, the frequency of IgG1^+^ B cells was approximately 50% of the WT level at each cell division. In addition, the percentages of proliferating B cells present at the 4th to 6th cell divisions were significantly reduced in the absence of PP4, whereas cells accumulated at the 2nd and 3rd cell divisions (Figs. 1c and 1d). Moreover, after 72 h of LPS + IL-4 stimulation, these PP4-deficient B cells showed a strong reduction in viability (Fig. 1e). We next examined cell cycle progression in vitro by measuring BrdU incorporation by resting and stimulated WT and mutant B cells, and found that PP4 deficiency induced cell cycle arrest in S phase (Figs. 1f and 1g). Thus, PP4 deficiency in activated B cells leads to defects in both cell proliferation and CSR.

### The ATM-p53 pathway is strongly induced in PP4-deficient B cells

**Fig. 2:**
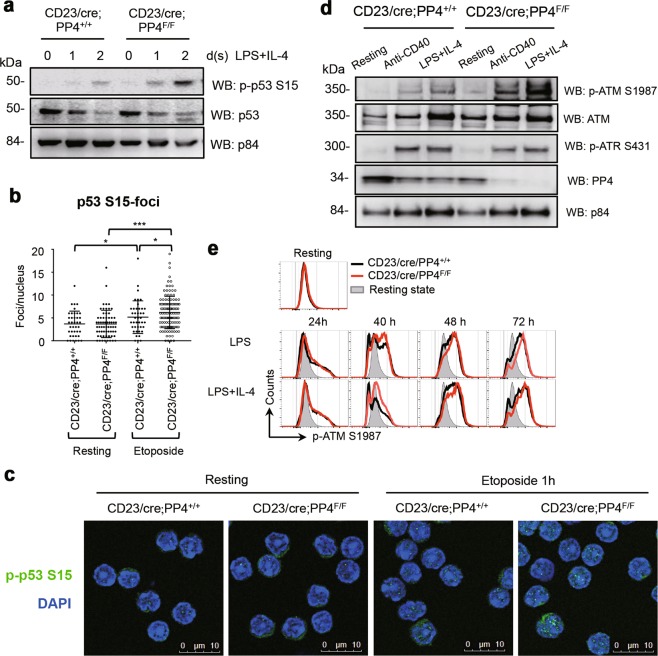
The ATM-p53 pathway is strongly induced in PP4-deficient B cells. **a** Immunoblots to detect phospho-p53 (*p*-p53) S15, total p53 and p84 (nuclear loading control) in the nuclear fraction of splenic B cells that were isolated from mice of the indicated genotypes (*n* = 3/group) and left unstimulated (0), or stimulated with LPS + IL-4 for 1 or 2 days. Data are representative of two independent trials. **b** Quantitation of the number of nuclear *p*-p53 S15-foci/cell in purified splenic B cells that were isolated from mice of the indicated genotypes and were left untreated (resting) or treated with 10 μM etoposide for 1 h in vitro. Data are values for individual mice (*n* = 3/group). Horizontal bars = mean values ± SD (cell number 39–126/group). *p ≤ 0.05. ***p ≤ 0.0005. **c** Representative confocal microscopy images of cells in the experiment described in **b**. Data are representative of two independent trials. **d** Immunoblots to detect phospho-ATM (*p*-ATM) S1987, total ATM, *p*-ATR S431, total ATR, and p84 in the nuclear fraction of B cells of the indicated genotypes that were left untreated (resting) or were stimulated with anti-CD40 or LPS + IL-4 for 4 days. Data are representative of two independent trials. **e** Curve overlay to show intracellular staining to detect *p*-ATM S1987 in B cells of the indicated genotypes that were left untreated (resting) or stimulated with LPS or LPS + IL-4 for the indicated times. Data are representative of two independent trials

To examine the consequences of PP4 deficiency in activated B cells at the signal transduction level, we performed immunoblotting to detect p53 phosphorylated at S15 (*p*-p53 S15) in extracts of control and CD23/cre;PP4^F/F^ B cells that had been stimulated for 1 or 2 days with LPS + IL-4 in vitro. Indeed, *p*-p53 S15 levels were highly elevated in the mutant B cells (Fig. 2a). In addition, increased numbers of both *p*-p53 S15-foci and p53-foci were observed in cultures of etoposide-treated PP4-deficient B cells compared to WT controls (Figs. 2b and 2c; Supplementary Fig. 1). In response to DNA damage, p53 phosphorylation at S15 is catalyzed by either activated ATM or ATR [48–50]. We found that PP4-deficient B cells stimulated with anti-CD40 or LPS + IL-4 showed higher levels of *p*-ATM S1987 but less *p*-ATR S431 than controls (Fig. 2d). This enhanced *p*-ATM S1987 signal appeared in the mutant B cells as early as at 40 h post-stimulation revealed by intracellular staining (Fig. 2e). These results suggest that, in response to LPS + IL-4, PP4-deficient B cells activate the ATM-p53 axis to trigger a vigorous DNA damage response.

### Induction of p53 facilitates CSR in PP4-deficient mature B cells by promoting cell survival

**Fig. 3:**
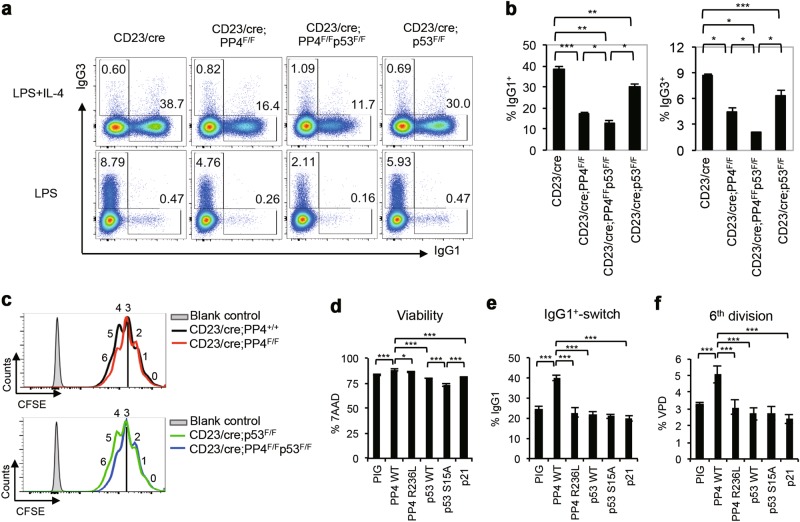
Induction of p53 facilitates CSR in PP4-deficient mature B cells by promoting cell survival. **a** FACS profiles of IgG1 versus IgG3 expression by B220^+^IgM^-^IgD^-^ gated B cells that were isolated from mice of the indicated genotypes (*n* = 3/group) and stimulated in vitro with LPS + IL-4 or LPS for 72 h. Data are representative of three independent trials. **b** Quantitation of the percentage of IgG1^+^- and IgG3^+^-switched B cells among total B cells stimulated by LPS + IL-4 (left) and LPS (right) from the data in **a**. Data are the mean ± SD (*n* = 3/group). **c** Curve overlay to show CFSE decay in B cells in (top) CD23/cre;PP4^+/+^ versus CD23/cre;PP4^F/F^, and (bottom) CD23/cre;p53^F/F^ versus CD23/cre;PP4^F/F^/p53^F/F^ cultures that were stimulated with LPS + IL-4 for 72 h. Numbers 0 to 6 indicate cell divisions. Data are representative of two independent trials. **d** Quantitation of percent viability as determined by 7AAD staining among GFP^+^ B cells that had been retrovirally transduced with pMSCV-PIG empty vector, or vectors encoding PP4 WT, PP4 R236L, p53 WT, p53 S15A, or p21, as indicated. Data are the mean ± SD (*n* = 4/group). **e** Quantitation of the percentage of IgG1-switched GFP^+^IgM^-^IgD^-^ cells among the transduced B cells in **d**. Data are the mean ± SD (*n* = 4/group). **f** Quantitation of %VPD-positive cells at the 6^th^ cell division among the transduced B cells in **d**. Data are the mean ± SD (*n* = 4/group)

To explore p53’s role in Ig class switching, we generated CD23/cre;PP4^F/F^p53^F/F^ mice and control littermates. We stimulated B cells from CD23/cre;PP4^+/+^, CD23/cre;PP4^F/F^, CD23/cre;PP4^F/F^p53^F/F^, and CD23/cre;p53^F/F^ mice with LPS + IL-4 in vitro and measured CSR efficiency. Double deficiency of PP4 and p53 increased the severity of the defects in IgG1^+^-switching and IgG3^+^-switching compared to loss of PP4 alone (Figs. 3a and 3b), suggesting that p53 induction in PP4-deficient B cells protects CSR.

We next applied CFSE labeling to our double mutant B cells and found that their proliferation was still compromised (Fig. 3c). We then performed retroviral transduction of B cells from CD23/cre;PP4^F/F^p53^F/F^ mice to achieve ectopic expression of either PP4 WT, PP4 phosphatase-dead mutant R236L [51], p53 WT, p53 S15A, or p21, and subjected these cells to stimulation with LPS + IL-4 for 72 h in vitro. Double mutant B cells transduced with PP4 showed increased viability compared to mock-transduced controls, whereas transduction of PP4 R236L, p53, or p21 impaired cell survival (Fig. 3d). Intriguingly, transduction of p53 S15A reduced cell viability more severely than transduction of p53 WT or p21, suggesting that the cell cycle arrest induced by the p53-p21 pathway contributes to mutant B cell survival. However, only double mutant B cells transduced with PP4 WT were able to undergo IgG1-switching and proliferate until the 6th cell division (Figs. 3e and 3f; Supplementary Fig. 2). These findings suggest that p53 activation in PP4-deficient B cells is beneficial for CSR because it promotes cell survival through induction of cell cycle arrest.

### Impaired ATR activation and reduced γH2AX-NBS1 complex formation in PP4-deficient B cells

**Fig. 4:**
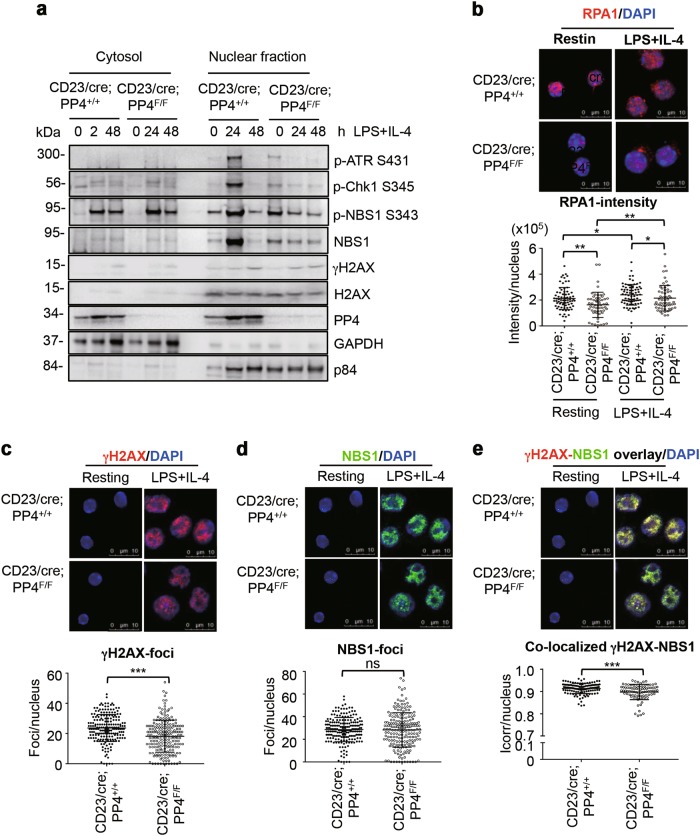
Deletion of PP4 by CD23/cre reduces CSR efficiency. **a** Immunoblots to detect *p*-ATR S431, *p*-Chk1 S345, *p*-NBS1 S343, NBS1, γH2AX, H2AX, PP4, GAPDH and p84 in the cytosolic and nuclear fractions of B cells of the indicated genotypes that were left unstimulated (0), or stimulated with LPS + IL-4 for 24 h or 48 h. Data are representative of two independent trials. **b** Confocal microscopy images of immunofluorescent staining to detect RPA1 and quantitation of the intensity of RPA1 stainining in B cells of the indicated genotypes that were left untreated (resting) or stimulated with LPS + IL-4 for 24 h. **c** Confocal microscopy images of immunostaining to detect γH2AX (red) and quantitation of γH2AX-foci in B cells of the indicated genotypes that were left untreated (resting) or stimulated with LPS + IL-4 for 48 h. **d** Confocal microscopy images of immunostaining to detect NBS1 (green) and quantitation of NBS1-foci in the experiment described in **c**. **e** Confocal microscopy images of immunostaining to detect γH2AX overlaid with NBS1 and quantitation of colocalized γH2AX-NBS1 foci (index of correlation, Icorr) in the experiment described in **c**. Horizontal line = mean ± SD. Data from **b–e** are analyzed by MetaMorph. Values are for individual cells, and representative of two independent experiments. ns, not significant

ATR is a master conductor of cellular responses to DNA replication stress. Upon activation, mouse ATR is phosphorylated at several residues, including S431 (equivalent to human ATR S428) [52, 53]. Activated ATR further phosphorylates Chk1 on S345, that induces cell cycle arrest [54]. To dissect the signaling events responsible for the proliferation defect in PP4-deficient B cells, we stimulated CD23/cre;PP4^+/+^ and CD23/cre;PP4^F/F^ B cells with LPS + IL-4 in vitro for 24 h or 48 h, and subjected cytosolic and nuclear lysates to immunoblotting to detect *p*-ATR S431. In control B cells, LPS + IL-4 stimulation induced ATR S431 phosphorylation on day 1, followed by a decrease in this phosphorylation signal by 48 h (Fig. 4a). However, PP4-deficient B cells did not induce *p*-ATR S431 signal upon stimulation for 24 h nor 48 h. Consistent with this, LPS + IL-4 induced Chk1 S345-phosphorylation in control cells on 24 h after stimulation, which was with the same kinetic as ATR activation. However, the inducible Chk1-phosphorylation on S345 on 24 h post stimulation was absent in PP4-deficient B cells (Fig. 4a). The results reveal that PP4 deficiency impairs the activation of ATR-Chk1 pathway.

During DNA damage, NBS1 and H2AX are phosphorylated by ATM or ATR and recruited to DNA damage sites [4] [55–57]. We therefore used immunoblotting to determine whether PP4 deficiency affected nuclear localization of NBS1 and γH2AX in activated B cells. In the resting status, a low level of NBS1 was detectable in the nuclear fraction of WT and PP4-deficient B cells. Upon 24 h after stimulation with LPS + IL-4, NBS1 became strongly phosphorylated on S343 and accumulated in WT nucleus. The *p*-NBS1 S343 signal dropped in WT nucleus at 48 h post-stimulation, which was associated with downregulation of total NBS1 protein (Fig. 4a). Although *p*-NBS1 S343 was detected in the cytosol of PP4-deficient B cells, the nuclear translocation of *p*-NBS1 S343 was severely blocked. These results suggest that PP4 is necessary for the nuclear translocation of NBS1 and normal activation of ATR-Chk1 pathway in response to signals triggering Ig class switching.

During DNA replication, RPA proteins are recruited to single-stranded lesions in DNA to stabilize the stalled forks and signal for ATR activation [9, 10]. In addition, a direct interaction between RPA1 and MRN complex has been demonstrated [58]. To address whether the impaired nuclear translocation of NBS1 in PP4-deficient B cells was associated with a reduction of RPA-foci formation, we performed immunofluorescent staining to detect nuclear RPA1 in CD23/cre;PP4^+/+^ and CD23/cre;PP4^F/F^ B cells that were left untreated or stimulated with LPS + IL-4 for 24 h. In resting control B cells, RPA1 was enriched in the nucleus and appeared as coarse particles (Fig. 4b**)**. Stimulation by LPS + IL-4 slightly increased the intensity of nuclear RPA1 staining, which appeared “cloudy” and more widely distributed than in unstimulated WT cells. Although the morphology of nuclear RPA1 in resting or stimulated PP4-deficient B cells was similar to that in the WT controls, the intensity of RPA1 staining in PP4-deficient B cells was much less in both cases (Fig. 4b). These findings indicate that PP4 deficiency in B cells reduces RPA1 recruitment, potentially weakening ATR activation.

The above results prompted us to use confocal microscopy to examine the effect of PP4 deficiency on γH2AX-foci induced in response to LPS + IL-4. Although PP4 has been demonstrated to function as a γH2AX-phosphatase that is required for the recovery from DNA damage [33–35], we did not observe any significant accumulation of γH2AX in PP4-deficient B cells in the resting status or on 24 h post stimulation with LPS + IL-4 (Fig. 4a). Due to mitotic effect driven by LPS, the appearance of H2AX in the cytosolic fractions, especially on 48 h post stimulation, reflected an increased amount of cells in prometaphase. Indeed, cultures of PP4-deficient B cells showed many fewer γH2AX-foci than cultures of WT B cells at 48 h post-stimulation with LPS + IL-4 (Fig. 4c). Although NBS1-focus formation was comparable between WT and PP4-deficient B cells, the co-localization of γH2AX-NBS1 signals was significantly reduced in PP4-deficient B cells at 48 h post-stimulation (Figs. 4d and 4e). Thus, PP4 deficiency in B cells reduces the retention of γH2AX-NBS1 complexes and impairs ATR-Chk1 activation on DNA damage sites.

### Deletion of PP4 by AID/cre does not impair CSR in vitro

**Fig. 5:**
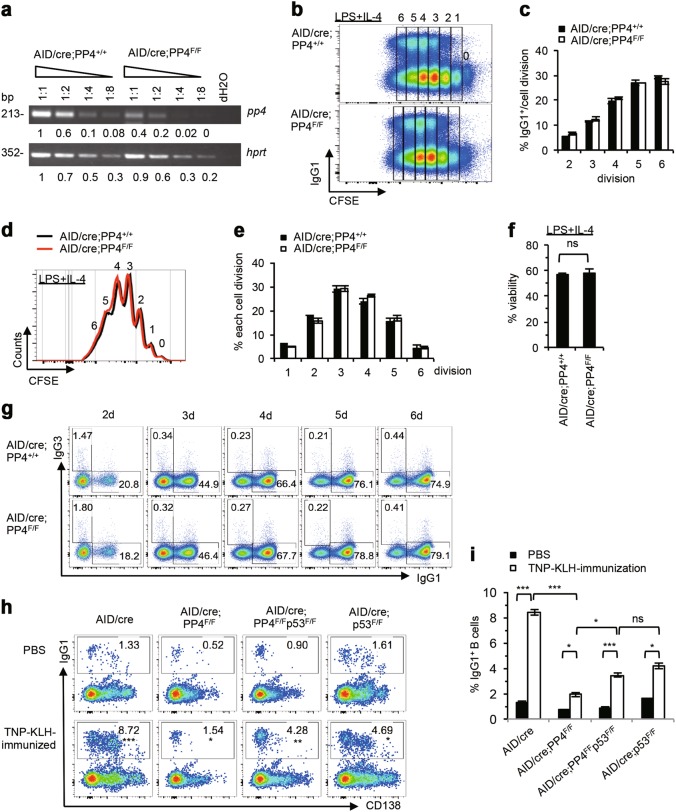
Deletion of PP4 by AID/cre restores B cell proliferation and IgG1-switching in vitro. **a** Semi-quantitative RT-PCR analysis of *pp4* mRNA expression in B cells of the indicated genotypes that were stimulated with LPS + IL-4 for 72 h in vitro. Extracts were diluted as indicated. *hprt*, loading control. Data are representative of two independent trials. **b** FACS profiles of CFSE decay versus IgG1 expression in B220^+^IgM^-^IgD^-^ gated B cells of the indicated genotypes that were stimulated with LPS + IL-4 for 72 h in vitro. Numbers 1–6 indicate cell divisions. Data are representative of three independent experiments. **c** Quantitation of IgG1-switched B cells at the 2nd to 6th cell divisions among the B220^+^IgM^−^IgD^−^ gated B cells in the experiment described in **b**. Data are the mean ± SD of triplicates. **d** CFSE overlay of proliferating B cells in the experiment described in **b**. Data are representative of three independent trials**. e** Quantitation of frequencies of proliferating B cells at the 1st to 6th cell divisions in the experiment described in **b**. Data are the mean ± SD (*n* = 4/group). **f** Quantitation of percent viability for the B cells in the experiment described in **b**. **g** FACS profiles of the kinetics of induction of IgG1 versus IgG3 expression by IgM^−^IgD^−^ gated B cells of the indicated genotypes stimulated with LPS + IL-4 for 2 to 6 days in vitro, as indicated. Data are representative of two independent experiments. **h** FACS profiles of CD138 versus IgG1 expression by IgM^-^IgD^-^ gated B cells that were isolated from mice of the indicated genotypes on day 14 post-immunization with TNP-KLH. Data are representative of two independent experiments. **i** Quantitation of the percentage of IgG1^+^ B cells in the experiment described in **h**. Data are the mean ± SD (*n* = 3–5/group)

Our data indicated that the defective CSR in PP4-deficient B cells was associated with their proliferation defect. We therefore investigated whether deletion of PP4 mediated by AID/cre in GC B cells, which affects the stage beyond cellular proliferation, could restore normal CSR efficiency. We generated AID/cre;PP4^+/+^ and AID/cre;PP4^F/F^ mice, purified splenic B cells from these animals, and examined IgG1-switching following LPS + IL-4 stimulation of these B cells in vitro. The efficiency of PP4 deletion induced by AID/cre was approximately 60% at 72 h post-stimulation (Fig. 5a). Within the first 6 cell divisions, B cells from AID/cre;PP4^+/+^ and AID/cre;PP4^F/F^ mice showed an equal ability to generate IgG1-expressing B cells (Figs. 5b and 5c). Examination of total B cell frequencies through the 1st to 6th cell divisions showed that the cell cycling of LPS + IL-4-stimulated AID/cre;PP4^F/F^ B cells was comparable to that of AID/cre;PP4^+/+^ controls (Figs. 5d and 5e), as was their viability (Fig. 5f). We then monitored the frequency of IgG1-switching in LPS + IL-4-stimulated B cells at 24 h intervals over 2 to 6 days of culture and found no differences between the genotypes (Fig. 5g). Thus, deletion of PP4 after the cell proliferation phase does not affect class switching in vitro.

To study the effect of AID/cre-induced PP4 deficiency on CSR in vivo, we immunized AID/cre;PP4^+/+^ and AID/cre;PP4^F/F^ mice with TNP-KLH emulsified in alum and compared the proportion of IgG1^+^ B cells present at 14 days post-immunization with that in PBS-immunized controls. In AID/cre;PP4^+/+^ mice, IgG1^+^ B cells increased from 1.3% to 8.7% after immunization (Figs. 5h and 5i). However, IgG1^+^ B cells were poorly induced in immunized AID/cre;PP4^F/F^ mice and only increased from 0.52% to 1.54%. We then generated AID/cre;p53^F/F^ and AID/cre;PP4^F/F^p53^F/F^ mice to investigate whether a double deficiency of PP4 and p53 mediated by AID/cre could influence IgG1-switching upon TNP-KLH-immunization in vivo. Remarkably, IgG1^+^ B cells in immunized AID/cre;PP4^F/F^p53^F/F^ mice rose from 0.9% to 4.2%, an almost 3-fold increase over the level of these cells in immunized AID/cre;PP4^F/F^ mice (Figs. 5h and 5i). These results show that deletion of PP4 by AID/cre completely rescues CSR in vitro, and that ablation of both PP4 and p53 by AID/cre can partially rescue IgG1-switching in vivo.

### Deletion of p53 promotes the production of germline transcripts

**Fig. 6:**
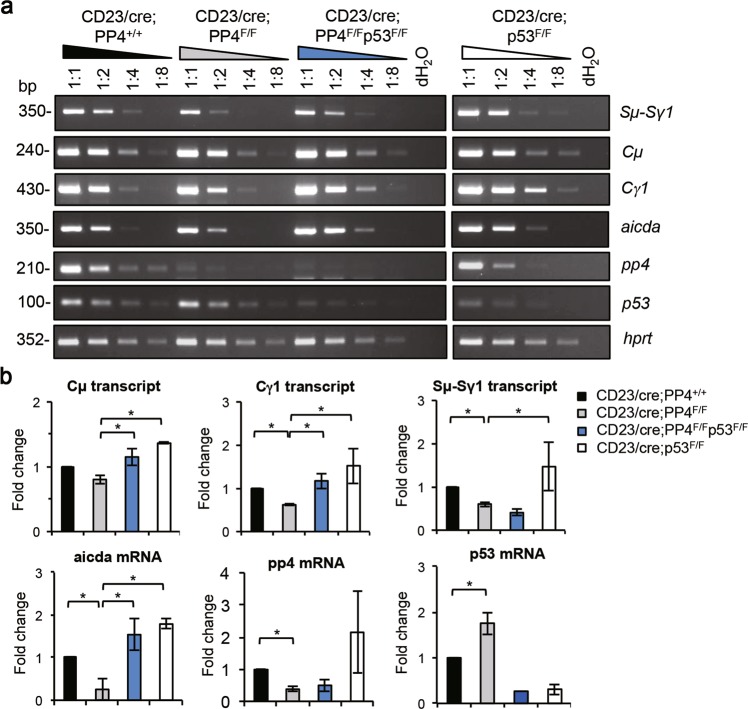
p53 deficiency restores normal levels of germline transcript Cγ1. **a** RT-PCR analysis of B cells that were isolated from mice of the indicated genotypes (*n* = 4/group) and stimulated in vitro with LPS + IL-4 for 72 h. Extracts were diluted as indicated and analyzed to detect switched transcript Sμ-Sγ1, germline transcripts Cμ and Cγ1, and *pp4*, *p53*, *aicda*, and *hprt* mRNAs. Data are representative of two independent trials. **b** Quantitation of fold change in the levels of the transcripts in the experiment described in **a**

The production of donor and acceptor germline transcripts is a prerequisite for CSR. To determine why p53 deletion rescued CSR in PP4-deficient B cells, we used semi-quantitative RT-PCR to determine levels of the Cμ, Cγ1, and Sμ-Sγ1 germline transcripts in B cells from CD23/cre;PP4^+/+^, CD23/cre;PP4^F/F^, CD23/cre;PP4^F/F^p53^F/F^, and CD23/cre;p53^F/F^ mice. In the same experiment, we measured relative mRNA levels of *aicda*, *pp4*, *p53*, and *hprt* (control) in these cells (Fig. 6a). Quantitative analysis of the relative expression of these transcripts revealed that levels of each transcript examined (except p53) were significantly reduced in the absence of PP4 alone (Fig. 6b). Notably, loss of both PP4 and p53 restored normal levels of germline transcript Cγ1 and *aicda* mRNA (Figs. 6a and 6b). These findings suggest that PP4 is required for normal germline transcript production, that p53 exerts a suppressive effect on CSR, and that inactivation of p53 promotes the generation of germline acceptor transcripts.

**Fig. 7:**
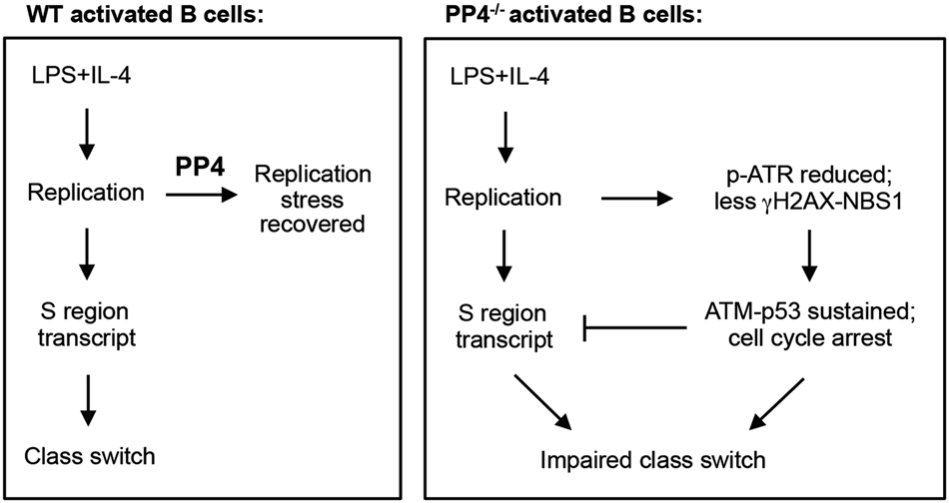
Schematic illustration of proposed roles for PP4 in B cell proliferation and Ig class switching. (Left) In WT activated B cells, LPS + IL-4 stimulation results in cell proliferation requiring DNA replication. In the presence of PP4, replication stress is prevented, S region transcripts are produced, CSR occurs with normal efficiency, and Ig class switching is normal. (Right) In activated PP4-deficient B cells, ATR activation is decreased and reduces the retention of γH2AX-NBS1 complexes on the DNA DSB sites needed for DNA replication and CSR. A sustained DNA damage response is triggered via the ATM-p53 axis that results in cell cycle arrest, promoting cell viability. On the other hand, p53 exerts a suppressive effect on the production of germline acceptor S region transcripts

Taken together, our data indicate that PP4 deficiency decreases ATR activation and thereby reduces the retention of γH2AX-NBS1 complexes on DNA damage sites, leading to p53 activation and a sustained DNA damage response (Fig. 7). However, deletion of PP4 after B cells have proliferated rescues CSR in vitro. In vivo, ablation of PP4 and p53 at the GC B cell stage by AID/cre partially rescues CSR through the increased sustained production of germline acceptor transcripts.

## Discussion

In this study, we demonstrate that PP4 is essential for the avoidance of DNA replication stress, whose prevention is a prerequisite for CSR. In response to LPS + IL-4, substances that induce class switching to IgG1, PP4-deficient B cells show a defect in cell proliferation due to cell cycle arrest in S phase, as well as reduced cell survival. We find that PP4 deficiency strongly reduces RPA1 intensity and affects the nuclear translocation of NBS1 upon the LPS + IL-4. ATR-Chk1 pathway is thus not well activated in LPS + IL-4-stimulated B cells lacking PP4, and the number of γH2AX-NBS1-foci retained at sites of DNA damage is decreased. It is likely that the reduced B cell proliferation and S phase arrest reflects the attenuated ATR signaling pathway in the absence of PP4. Severe DNA damage accumulates that then induces strong activation of the ATM-p53 pathway. However, when PP4 is deleted by AID/cre at GC B cell stage, which is beyond the cell proliferation phase, IgG1-switching is completely restored to normal in vitro. In vivo, however, genetic ablation of PP4 by AID/cre fails to rescue class switching. We have previously shown that PP4 deficiency affects BCR signaling and antigen-specific clonal expansion in vivo [42]. Thereby, we speculate that this defect in IgG1-switching is an indirect consequence of the impaired GC formation evident in immunized AID/cre;PP4^F/F^ mice (data not shown) because CSR takes place late during GC formation. However, we found that deletion of both PP4 and p53 by AID/cre in vivo partially restored the frequency of IgG1^+^ B cells in mutant mice immunized with TNR-KLH. Interestingly, most of the IgG1^+^ B cells isolated from these double mutant mice lack CD138 expression, suggesting that their differentiation occurs in a GC-independent manner in vivo.

It is notable that LPS + IL-4 stimulation induced the ATM-p53 pathway modestly in WT B cells but dramatically in PP4-deficient B cells. Genetic ablation of both PP4 and p53 by CD23/cre did not rescue the Ig class switching defect apparent in B cells lacking PP4 alone, suggesting that PP4 deficiency impairs DNA replication via a p53-independent mechanism. Nevertheless, double deficiency of PP4 and p53 in B cells mediated by CD23/cre significantly promoted the production of germline acceptor S region transcripts, and the level of these transcripts is an important determinant of class switching [59]. These results suggest that p53 acts as a gatekeeper controlling the level of germline acceptor S region transcripts. Taken together, our study indicates that p53 plays a dual role in CSR in PP4-deficient B cells. On one hand, p53 sustains the viability of PP4-deficient B cells through p21-mediated cell cycle arrest, which is beneficial for the repair of DSBs induced during DNA replication. On the other hand, p53 stabilization in B cells exerts a suppressive effect on the production of germline acceptor S region transcripts.

H2AX and NBS1 act synergistically in the HR pathway of DNA repair [12, 13]. The accumulation of NBS1-γH2AX complexes on DNA damage sites is essential for the repair and rejoining of DSBs, a process that is coordinated with the induction of cell cycle arrest [60]. NBS1 is phosphorylated at S343 by ATM in response to radiation, an event that mediates an S phase checkpoint [55–57]. Mutation of NBS1 S343 disturbs this S phase arrest and reduces DNA repair by HR [61]. We find that, in B cells where PP4 deficiency is mediated by CD23/cre, *p*-NBS1 S343 is slightly accumulated in the cytosolic fraction of cells at 24 h post-stimulation by LPS + IL-4, suggesting that the S phase checkpoint imposed by ATM-NBS1 is activated in PP4-deficient B cells. In addition, the cytosolic retention of *p*-NBS1 S343 is associated with a dramatic reduction of nuclear NBS1 in PP4-deficient B cells. PP4 has been shown to dephosphorylate RPA2, which is necessary for efficient HR-mediated DNA repair [36]. Abolition of RPA2 phosphorylation impedes the loading of the essential repair factor RAD51 and extends the G2-M checkpoint. Interestingly, we show that loss of PP4 strongly reduces RPA1-intenstiy in resting and LPS + IL-4-stimualted B cells, suggesting that the RPA complex in the absence of PP4 is defective in the complex assembly. Whether the nuclear translocation of NBS1 is directly regulated by PP4, or is an indirect effect mediated by RPA disassembly, requires further investigation.

Our finding implies that PP4 deficiency in B cells affects the sequential recruitment of RPA and NBS1, leading to an impaired ATR signaling pathway, which is required for cell proliferation. Due to the key roles of NBS1 and γH2AX in CSR, the reduction in NBS1-γH2AX complexes exerted by PP4 deficiency further decreases CSR efficiency. Our study therefore establishes that PP4 is a key component required for efficient recruitment of NBS1 and γH2AX during B cell proliferation and Ig class switching.

## Electronic supplementary material

Supplemental Results
