# Supplementary material for: PP4 deficiency leads to DNA replication stress that impairs immunoglobulin class switch efficiency
Source: Cell Death Differ. 2018 Sep 20;26(7):1221–34. doi: 10.1038/s41418-018-0199-z (PMC6748143; doi:10.1038/s41418-018-0199-z)
Supplement: Supplementary file 1 — Supplemental Results [file 41418_2018_199_MOESM1_ESM.docx]

**Supplementary Figure 1**

**
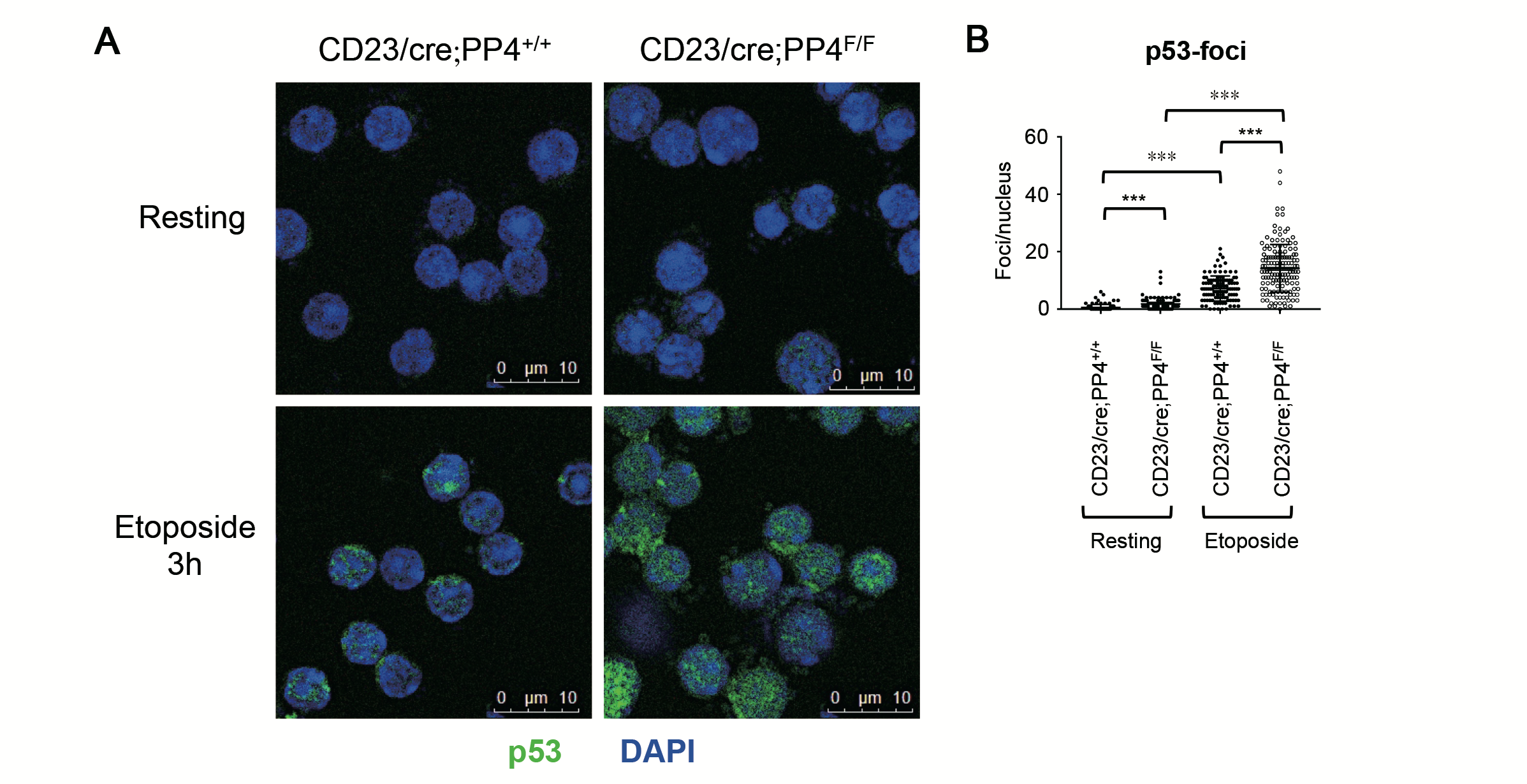
**

**Increased p53-foci in PP4-deficient B cells treated with etoposide. (A)** Confocal microscopy images of anti-p53 staining versus DAPI in purified splenic B cells of the indicated genotypes that were left untreated (resting) or treated with 10 μM etoposide for 3h. **(B)** Quantitation of numbers of p53-foci in the experiment described in (A). Data are values for individual cells (n=3/group). Horizontal line = mean value ± SD (cell number 95-156/group). ***, p ≤ 0.0005.

**Supplementary Figure 2**

**
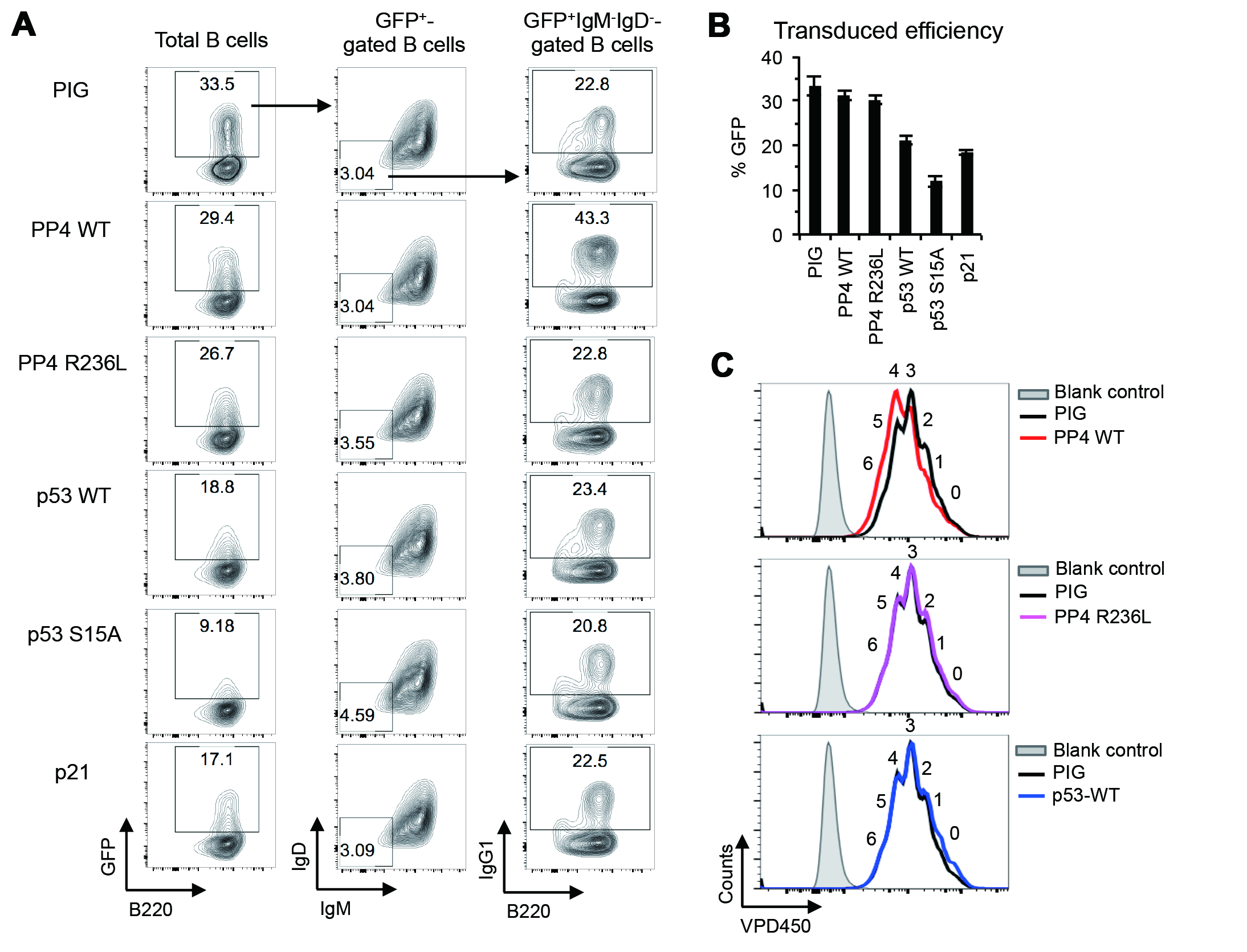
**

**Transduction of PP4 WT into B cells from CD23/cre;PP4^F/F^p53^F/F^ mice restores cell proliferation. (A)** Flow cytometric analyses to confirm retroviral transduction of PP4 WT, PP4 R236L mutant, p53 WT, p53 S15A mutant and p21 in pMSCV-PIG empty vector encoding GFP. B cells from CD23/cre;PP4^F/F^p53^F/F^ mice were first labeled with fluorescent dye VPD450, stimulated with LPS+IL-4 for 24h, and transduced with PIG vectors co-expressing the genes of interest as well as GFP, as indicated. After 48h, cells were subjected to flow cytometric analysis. The frequencies of IgG1^+^ cells were calculated from GFP^+^IgM^-^IgD^-^ B cells, as indicated by the arrows. **(B)** Quantitation of % GFP^+^ cells as a measure of transduction efficiency in the experiment described in (A). Data are the mean ± SD (n=4/group). **(C)** Curve overlays to show B cell proliferation as measured by VPD450 decay in GFP^+^ B cells transduced with PIG (black) and overlaid with PP4 WT (red, upper panel), PP4 R236L (pink, middle panel) or p53 WT (blue, lower panel), as described in (A). Numbers indicating cell divisions from resting (0) to the 6^th^ cell division are shown.
